# Dr. Otto Ringk’s Theory and Treatment of Diphtheria

**Published:** 1886-02

**Authors:** 


### DR. OTTO RINGK'S THEORY AND TREATMENT OF DIPHTHERIA,

*Translated from the German by F. A. Schmidt, M. D., Schulenburg, Texas.*

For Daniel's Texas Medical Journal.

IN a late number of one of the many periodicals published in the German capital, I find an interesting article on diphtheria, by Dr. Otto Ringk. As the nature, etiology and treatment of this dreadful scourge—as given in the paper—is peculiarly novel, I thought it worth while to translate the main points of said article, and give them to your readers for what they are worth. Americans are conceded to be a practical people, having but little of the pedantry peculiar to some of the older nations, and if any *practical* results can be derived, we will have them.

Dr. Ringk is an adherent of the morbid germ theory as cause of most diseases—but, unlike the originators of this theory, invests a peculiar set of germs (monades) with the power of being instigators of diseases for which we hypothetically have several species.

He (Dr. R.) is a disciple of the late Dr. C. Huster, who, in 1868, widened the discoveries of Pasteur relative to the germs of putrefaction, also into *morbid* germs of living tissue.

He very concisely explains the changes produced by these micro-organisms after having gained access into the body and blood of the living, and shows experimentally the alteration the blood corpucles undergo if subjected to the action of these germs. The microscope, he says, then shows them (the blood corpucles) to have changed their rounded form to a mulberry (barbed) shape, and he explains the necessary pathological consequence of this.

He avers, that after having pierced the capillaries, the lymphatics, and through these channels having entered the general circulation, they thrust themselves into the white and red blood corpucles—multiplying enormously—and begin their pernicious influence by changing the form of the blood corpucles, as before explained.

This change of form, depriving them of their former elasticity, perniciously affects the function of those bodies, (the blood corpucles). He says that they, in this condition, must necessarily become entangled among themselves, fail to pass through the capillaries, and these in consequence become clogged—the blood following—by the impetus of the heart's acaction—can not advance properly, and is subjected to a similar disastrous inactivity; and lastly the larger vessels fail to serve as channels for the conveyance of the life-giving fluid.

The Doctor testifies to having been present when Dr. Huster and Dr. Ludwig, of Leipsic, conducted experiments to prove that a large part (one half) of the blood of an animal could, in this way, be made stationary.

He supposes that the sometimes observed suddenly occurring fatal diphtheric cases in patients in whom the diphtheric process in the throat gave no cause for immediate alarm, are caused in this way, giving rise (being the cause of) to apoplexy of heart or brain.

He then illustrates their (the monades) effect on the human economy by three imaginary cases as follows :

Three persons in sound health and under the same conditions, accidently acquire a simple wound (cut). Notwithstanding the simplicity and similarity in all respects, of traumatism and individuals, the healing process will (may) be different in each person. The edges of the wound of one will soon adhere, be covered by a crust, and may in fact, heal without any inconvenience to the person whatever, (by first intention). With the second individual the healing process is not quite as simple; the edges of his wound will also adhere, but not as perfectly as with the other; pus will be formed beneath the crust; still in a few days the wound will be healed, (by granulation).

Quite different, indeed, may become the condition of the third patient. Shortly after the accident, the edges of his wound begin to redden, become swollen, painfully inflamed ; constitutional symptoms (fever) set in ; the inflammation rapidly spreads to surrounding tissue, and attains a livid, malign hue (erysipelas). The near lymphatics become painfully enlarged, suppurative process may supervene in them, and our patient is now in iminent danger, and the case may even prove fatal.

Dr. Ringk then mentions the *natural* gateways for these germs into the human body, i. e., nose, mouth, pharynx, and the lungs during respiration. He explains what means nature adopts to ward off these germs, by ciliated epithelium and escaping fluids—and points to the pharynx *and the tonsils* as the least qualified to guard against their introduction in consequence of the many follicles in them—secreting no escaping fluids and interrupting the protecting epithelium. We know their (the follicles) orifices to form depressions well adapted for the harboring of morbid germs, and the natural warmth and moisture always present in these localities to tend to mature them.

The most simple cases of "monaded follicles," the writer says, are those in which a fit of coughing expels a small lump of a yellow pus-like substance, often the cause of alarm to patients and inexperienced practitioners. Aggravated cases of the same, the Doctor asserts, are cases of tonsilitis—simple and suppurative—the peculiar condition of a granulating surface checking the further advance of the invading germs.

He compares these cases (tonsilitis) to his second imaginary traumatic case, as he does his simplest case of "monaded follicle" to his first. Describing the worst cases—answering to his third traumatic case—he says, that the invading monades, contrary to those infecting but few follicles of the pharynx, have peculiar propagating faculties, and on account of increased mobility, a potent ability to thrust themselves inward. In glowing terms he describes these cases thus:

A few micro-organisms from the outside world deposited within the depressions of the follicular orifices, soon become the parents of new generations. Comparable to a well disciplined army, millions of monades march and countermarch on their chosen battlefield—the tonsils—and soon cover the entire surface of these glands; they irritate their tissue, causing the escape of white blood corpucles and fibrin. Monades, blood corpucles and fibrin unite to form a felt-like membrane covering the tonsils and surroundings. Other monades, situated directly beneath this membrane, work themselves incesantly further on, deeper and deeper, causing the well known tenacious adherence of the membrane to the tissue underneath. The forming and growing of this membrane is considered the most visible symptom of a disease now having become a pure case of diphtheria in its commencing stage.

"We have now arrived," the Doctor eloquently continues, at that disease, the very name of which fills every parent's heart with fear and apprehension, but no matter how near the danger is, the mask of mysteriousness has been torn from it; we are brought face to face with this dreaded pestilence, and we will be able to successfully combat the same as soon as we can convince every parent that the greatest danger in diphtheria is in *neglecting to call medical aid in time*, and as soon as every physician can be persuaded to look upon suspicious cases of *diseased tonsils* or the *nasal cavities* as *diphtheric in character.*"

"I have," the Doctor says, "adopted this course a number of years with the most gratifying results, not having lost a single case to which I was called in time;" and he challenges his patrons to contradict this assertion.

My own experience verifies this assertion:

The cases of diphtheria successfully treated by me, were those in which I was called early. In these cases my treatment remained *local* to the last. It consisted in *applying to the affected parts*—including the nasal cavities—a *saturated* solution, in hot water, of tannin and chlor. potass., as often as the membrane formed, which would generally but require a few hours; each application would effectually and *visibly* destroy the false membrane on tonsils and surroundings.

Further on, the Doctor says: "A number of physicians of our city (Berlin) are inclined to change the name of this disease to a more appropriate one, one more in accord with exact investigation and more in conformity with this theory" (monade theory), but he objects to this, for the reason that it would tend to confuse the public, who would be apt to presume a new disease, and be liable to fall back to the old habit of seeking medical aid at too late a period. "The public," he exclaims, "expect cure, not names."

His investigations, observations and deductions therefrom, induce him to proclaim and to culminate in the maxim "that diphtheria is nothing more nor less than *local putrifaction of*

*living tissue*, instigated by the introduction and consequent invasion of the putrifying germs of Pasteur," the fœtid exhalations of such diseased structures in themselves tending to prove the correctness of this assertion.

He, however, admits the existence of monades of increased virulence, having gained this virulence while acting in their simple capacity as putrifying ferments through local or other to us unknown influences.

The same he thinks may be true of the cholera bacillus, it being, perhaps, but a virulent form of the germ producing diarrhœa, cholera morbus, or cholerine.

He relates an instance in which he has been enabled to *fix* the sudden appearance of diphtheria in a family—three adults and one child—in the suburbs of Berlin, *directly* to decaying animal matter in the vicinity of their dwelling, the removal of which at once arrested the further spread of the disease. He then calls our attention to the opinion of Prof. Virchow, expressed years ago, to the effect that the pathological processes of hospital gangrene and diphtheria were similar in character.

Dr. Ringk declares both processes to be identical.

The gentleman will not be sustained in this respect by any one of the profession who had much experience with hospital gangrene, for several reasons, the least of which, in my estimation, is that isolation of hospital gangrene cases have proven to arrest the extension of the disease *in every instance.*

All wounded would not only be exceedingly liable to contract the disease, but would inevitably fall a prey to these germs, as they are known to exist everywhere and under all circumstances in the surrounding air. It is well known, however, that hospital gangrene appears primarily among the wounded in exceptional cases, and that these are the sources of infection of others.

Again, would such close relationship not imply that diphtheretic cases should sometimes develop among hospital gangrene patients, or those coming continually in close contact with them ?

During our late war I have had considerable experience with this "army pestilence," but I failed to observe the development of diphtheria in conjunction with hospital gangrene at any time which, with some reason indicates at least the specific nature of the hospital gangrene germ.

He maintains further, that all forms of erysipelas are attributable to "monades having nomadic proclivities" infesting the skin; he being able to arrest the onward march, or rather, change the course of the germs *in every instance* by hypodermic injections of dilute carbolic acid; in other words, to cure every case of erysipelas by these means; and gives the public a valuable hint (but by an objectionable procedure) in regard to arresting (curing) erysipelas of every and even the worst forms, by applying to the diseased surface pix liquida, renewed every three hours.

His treatment of diphtheria is not quite as simple as his theory is novel.

He uses antiseptics in the form of turpentine and mur. tr. of iron. He says:

"With no remedy have I been so successful in combating the initiatory diphtheritic fever, caused by the entrance of 'monades' into the circulation—as I have been with the oil of turpentine."

"After having diagnosed a case as diphtheria, I administer *immediately* a dose of ol. terebinth, one tablespoonful to an adult; children in proportion. If, after the administration of this dose, the fever does not subside, or the forming of diphtheritic membrane continue, I repeat the dose every three to six hours until the desired end is accomplished, or an effect is produced on the kidneys—difficulty in micturation or largely increased or diminished quantity of urine let. The prevailing idea of the evil effects of this drug on the kidneys, he says, has been greatly over estimated; my experience being, that children especially will bear this remedy well, and better than adults; it being but necessary to closely watch its efforts as stated, and no fear or apprehension in this respect need be entertained. Besides the turpentine (he continued) I am in the habit of prescribing the following mixture:

℞ Liq.ferr.sesqui chlo.................... 3.0
Aqua destil.............................9.00
Glycerine..................................1.00, M.S.,

From a desert to half tea spoonful, according to age of patient, every two hours; both mixtures, besides, alternately to be applied to the diseased parts with a camel-hair brush four to six times in the twenty-four hours. The nasal cavities also to be thoroughly disinfected by a mixture of turpentine eight parts and olive oil ninety parts, applied in such a way as to bring the entire surface of these cavities in contact with this disinfectant; he accomplishes this by dropping a quantity of the oil into the nose and giving the head such a position as to allow the oil to gravitate downwards and through the posterior nares into the throat.

